# Teacher Characteristics, Knowledge and Use of Evidence-Based Practices in Autism Education in Ireland

**DOI:** 10.1007/s10803-021-05223-1

**Published:** 2021-08-18

**Authors:** Lorna Barry, Jennifer Holloway, Stephen Gallagher, Jennifer McMahon

**Affiliations:** 1 i-TEACH Lab, Department of Psychology, University of Limerick, Limerick, Ireland; 2 School of Psychology, National University of Ireland Galway, Galway, Ireland; 3 Department of Psychology, Health Research Institute, University of Limerick, Limerick, Ireland

**Keywords:** Autism, Evidence-based practice, Research-to-practice, Teacher preparation

## Abstract

Autism evidence-based practices (EBPs) are those with demonstrated improved outcomes for students with autism across a range of skill areas, yet issues persist in adopting these in classroom settings- particularly in general education (GE) settings. This research aimed to identify teacher training, years of experience, access to allied professionals and knowledge and use of autism EBPs in GE settings in Ireland. 369 mainstream primary school teachers reported their characteristics and their knowledge and use of EBPs. Results indicated that the majority of teachers received little initial teacher education training in autism, almost no continuous professional development (CPD) before educating a child with autism, and received little support from allied professionals. Knowledge and use of EBPs differed significantly across teacher characteristics, with findings discussed in relation to teacher training.

In the field of autism education, a research-to-practice gap has been identified, whereby practices proven efficacious in the literature fail to be implemented in educational settings (Cook et al., 2009). Autism is a neurodevelopmental disorder, where individuals with the diagnosis present with deficits in social and communicative functioning (American Psychiatric Association, 2013). Children with autism often find it difficult to make friends and navigate the social environment of school (Rowley et al., 2012), may struggle academically due to challenges with language and concentration (Levy et al., 2010; McLean et al., 2014), and may not be able to keep abreast of the curriculum (Anglim et al., 2018). Students with autism are increasingly educated in general education (GE) classrooms by general educators who may not specialise in autism alongside their neurotypical peers (National Council for Special Education [NCSE], 2016). Within GE settings, students with autism may also receive support from special educators/ learning support teachers who often withdraw students for individualised instruction in small group settings, or may be educated in small-group autism special class settings (NCSE, 2016). As the majority of students with autism are now educated in these more inclusive education settings (Hehir et al., 2016), this necessitates that all teachers be able to flexibly meet the needs of a variety of students. The extent to which the teacher is able to provide individualised quality education has been found to be one of the most important factors influencing the outcomes of students with autism (Kasari & Smith, 2013), and even more able students with autism often require individualised support in the form of specific interventions (McMahon & Cullinan, 2016).

Interventions can typically be separated into evidence-based and unsupported practices (Paynter et al., 2017). Evidence-based practices (EBPs) for specific use with children with autism have been identified through rigorous reviews of the literature and most recently summarised by the National Clearinghouse on Autism Evidence and Practice (NCAEP) in the United States which has identified 28 such practices (Steinbrenner et al., 2020). These practices have been associated with improved outcomes for students with autism across social, communicative and academic functioning domains among others (Odom et al., 2010; Steinbrenner et al., 2020; Wong et al., 2015); and the use of EBPs with fidelity may also reduce teacher burnout (Ouellette et al., 2019). Furthermore, in education settings, the adoption of these EBPs is particularly important considering school-age children with autism spend the vast majority of their time in school (Brookman-Frazee, et al., 2009; Kasari & Smith, 2013).

Though there are positive outcomes associated with using EBPs, a research-to-practice gap in the autism EBP field has been identified (Cook & Odom, 2013). This discordance does not exist in a vacuum and is reflective of issues within the broader educational field, whereby no framework exists to support the systematic translation of research into practice (McGann et al., 2020). This discordance between research and practice has been particularly salient in the special education field, where specific interventions exist for teachers to use to support their students with special educational needs, yet these remain under-utilised (Burns & Ysseldyke, 2009; Cook & Odom, 2013). However, while there are broader issues of the contextual appropriateness of evidence-based practice undoubtedly at play, autism EBP research is unique in that a clear, well-defined set of EBPs has been developed by researchers over the last decade (Odom et al., 2010; Steinbrenner et al., 2020; Wong et al., 2015), providing teachers with a framework of reference from which to choose and employ EBPs. Furthermore, children with autism represent one of the most vulnerable school groups for whom EBPs can profoundly improve outcomes and as such translation of effective practices is vital.

The adoption of autism EBPs in school settings may be impacted due to the complex nature of schools (Kasari & Smith, 2013), as paradoxically, many interventions recommended for use in the school setting are developed and tested in clinical/1:1 settings. Thus, the school context where teachers have to teach larger groups of children, where the focus is usually placed on teaching academics, and where teachers typically already have a curriculum to implement, means that EBPs may be more challenging to adopt (Kasari & Smith, 2013); and research has found that teachers lack confidence implementing EBPs with their students with autism and find it difficult to select which practices to use (Brock et al., 2014; Anglim et el., 2018; Finlay et al., 2019). Existing literature has demonstrated that autism EBPs are not adopted frequently by GE teachers in GE settings. For example, recent research found that 90% of American special educators working in small group settings used EBPs such as reinforcement and modelling daily, whereas only 20% of general educators working in large group settings used these EBPs daily (McNeill, 2019). Furthermore, Australian research examining general educators use of EBPs found that they used only one EBP every day (visual supports), six EBPs “some of the time” and the remaining 20 EBPs were used “rarely” or “never” (Sulek et al., 2019).

To overcome the research-to-practice gap it is important to identify factors that can increase teachers’ use of autism EBPs (Barry et al., 2020a). Research has identified that teacher knowledge and training in EBPs is key to implementing EBPs with students with autism (Sulek et al., 2019), which is reflective of the broader autism EBP literature (Barry et al., 2020a; Paynter et al., 2017). An important facilitator of the implementation of EBPs may be support from allied health professionals such as psychologists, speech and language therapists and occupational therapists (Barry et al., 2020a), but research on this is limited to qualitative research and further exploration is warranted. Additionally, it is unclear in the literature the extent of training required to improve teachers EBP adoption, the impact of support on teachers’ use of EBPs, and if specific cohorts of teachers (e.g. general educators or special educators) may need to be targeted for training and support. Furthermore, there is a dearth of information available to inform our understanding of autism EBP adoption in Europe (Salomone et al., 2016), and we could identify no studies examining autism-specific EBP use in GE schools, nor the contextual factors which may impact their use. Given that European educational systems differ significantly from systems in other jurisdictions, such as the USA and Australia (McMahon & Cullinan, 2016), research that broadens understanding of the knowledge and implementation of autism EBP’s in a range of educational contexts as well as further examining implementation factors is a pressing need.

In sum, considering that outcomes for students with autism can improve through the use of EBPs (Steinbrenner et al., 2020), it is imperative that factors which bridge the research-to-practice gap evident in autism education are further explored. Preliminary evidence exists to support differences in EBP adoption by teachers based on a number of factors such as knowledge, training and support, but gaps remain in understanding how these factors impact in small group or large group settings, and furthermore, relatively few studies examining teachers’ adoption have been conducted outside of the USA (Barry et al., 2020a), meaning that contextual differences may exist which need to be delineated. To address the gaps in our knowledge, this paper aims to do the following:

1. Provide an overview of teacher characteristics in autism education such as types and intensity of training received, years’ experience, levels of education, levels of professional support received and knowledge and use of autism EBP’s.
2. Examine if knowledge and use of EBPs are associated with differences in teacher characteristics.

## Method

### Inclusion and Exclusion Criteria

Participants were first screened for inclusion, and if they met the criteria of, (a) being a mainstream primary school teacher, and (b) had experience teaching at least one child with autism, they were permitted to proceed through the online questionnaire.

### Participants Characteristics

Participants were 369 mainstream primary school teachers from all 26 counties in the Republic of Ireland, and comprised of 207 general educators, 62 special educators, 18 learning support teachers, 28 dual principal-teachers and 43 autism class teachers. Please see Table 1 for further participant characteristics.

**Table 1:** Participant demographics

Characteristic	Number (%)
Gender	
Male	44 (12.2%)
Female	311 (86.1%)
Prefer not to say	6 (1.7%)
Age Range	
20–25	35 (9.7%)
26–30	62 (17.1%)
31–35	66 (18.2%)
36–40	81 (22.4%)
41–45	47 (13%
46–50	21 (5.7%)
51–55	28 (7.7%)
56–60	18 (5%)
60 +	4 (1.1%)
Disadvantaged School*	65 (33%)
Rural School	135 (52.7%)

*Designated as a Delivering Equality of opportunity In Schools (DEIS) School. These are schools identified by the Department of Education and Skills in Ireland in which children are at greatest risk of educational disadvantage (Department of Education and Skills, n.d.)

### Sampling Procedures

Recruitment took place online via email and social media. Using the Department of Education and Skill’s school registry, 3105 schools were contacted to participate in the study by following an anonymous link to the survey provider Qualtrics. The anonymous link was also shared on the social media platforms Teachers’ Research Exchange (t-rex.ie), Facebook and Twitter. Due to the nature of anonymous online data collection, it is not possible to determine the complete response rate as we have no way of measuring how many people had access to the survey link.

### Design

This was a cross-sectional study. The dependent variables in this study were teachers use of EBPs and knowledge of EBPs. Independent variables included teacher position, education level, years of autism experience, autism specific training, school characteristics and support from external professionals.

### Measures

A self-report online survey was used to collect data. The first part of the survey collected demographic information as reported in Table 1. Teachers were asked questions related to their autism teaching experience and training, including their years of experience, training in their initial teacher education (ITE), and continuous professional development (CPD) training. Finally, teachers were asked about the support they had received from professionals and the number of times per year they had received support.

#### Knowledge and Use of EBPs

Teacher’s knowledge and use of EBPs was measured using an adapted version of the *Early Intervention Practices Scale* (Paynter & Keen, 2015). Some minor changes were made to this scale to make it contextually appropriate, for example, “parent-mediated interventions” and “pivotal response training” were removed, and speech and language therapy programmes and traditional teaching methods were added. A total of 37 interventions were included in the survey instrument. 27 of these interventions were designated EBP status, whilst 10 were unsupported practices. Participants rated their knowledge and use of interventions when educating students with autism on a five-point Likert scale from “very little” to a “very great extent” of knowledge, and from “never” to “frequently” use. Internal reliability for the scales remained high following minor amendments, Cronbach’s α for knowledge of EBPs scale = 0.97, Cronbach’s α for use of EBPs scale = 0.94.

### Procedure

Prior to distribution of the survey, two teachers took part in user experience testing of the survey, and a number of contextual adaptations were made to make the survey more aesthetically pleasing and socially valid. For example, the teachers were more familiar with the term “social stories” than social narratives, so “social stories/ social narratives” was included as an item in the survey. Once the survey was finalised, it was distributed via an anonymous survey link. Teachers who met the inclusion criteria could proceed to complete the survey which took an average of 12 min to complete.

### Data Availability and Data Analysis

The dataset analysed during the current study is available in the Zenodo repository (Barry et al., 2021). To address the first research question, descriptive statistics were conducted to identify teacher position, education level, years’ experience with children with autism, autism specific training, school characteristics and support from external professionals. To address the second research question, a series of One-Way Analysis of Variance (ANOVAs) and Independent samples t-tests were conducted, comparing teachers knowledge and use of EBPs across a number of teacher characteristics.

## Results

### Data Screening

Data were collected online via Qualtrics software and downloaded to IBM SPSS Statistics for Windows, Version 26 for analysis. Prior to analysis, the data were screened for survey completion, outliers and normality. Data were distributed normally and no outliers were identified. Missing value analysis conducted on the individual items revealed variable non-completions at the item level (4.3–43.6%). Data were missing completely at random (Little’s MCAR test: χ^2^ (42) = 27.33, *p* = 0.96), and thus list-wise deletion was used.

### Teachers Knowledge and Use of EBPs

Teachers were most knowledgeable (*M* = 4.00, *SD* = 1.20) about traditional teaching methods, and this was the only practice in which teachers scored “a great extent” of knowledge. Other practices which teachers had a moderate extent of knowledge of included visual supports (*M* = 3.96, *SD* = 1.10), social stories/ social narratives (*M* = 3.59, *SD* = 1.09), exercise (*M* = 3.29, *SD* = 1.14), modelling (*M* = 3.16, *SD* = 1.38), prompting (*M* = 3.02, *SD* = 1.30), reinforcement (*M* = 3.36, *SD* = 1.19), social skills training (*M* = 3.18, *SD* = 1.28) and sensory rooms (*M* = 3.33, *SD* = 1.27). The data indicates that teachers are knowledgeable to a moderate extent of a number of EBPs including visual supports, social stories/ social narratives, exercise, modelling, prompting, reinforcement, and social skills training (Steinbrenner et al., 2020). Overall, teachers’ knowledge of autism practices, both EBPs and unsupported practices appeared low, with 28 of 37 practices falling in the “very little” or “slight” category of knowledge.

Participants reported using six practices at least some of the time (mean score of 3 or above); exercise (*M* = 3.34, *SD* = 1.24), modelling (*M* = 3.16, *SD* = 1.33), reinforcement (*M* = 3.41, *SD* = 1.28), sensory rooms (*M* = 3.18, *SD* = 1.42), social stories/ social narratives (*M* = 3.44, *SD* = 1.21) and traditional teaching methods (*M* = 3.35, *SD* = 1.34), with four of these practices (exercise, modelling, reinforcement and social stories/ social narratives) meeting criteria for EBP (Steinbrenner et al., 2020). The most used practice was social stories/ social narratives (*M* = 3.44, *SD* = 1.21). Only two EBPs were used by over half of the teachers daily- reinforcement and social stories/ social narratives.

There were 14 rarely used practices (1 = rare); ABA, DRI/A/O, discrete trial training, extinction, facilitated communication, functional behaviour assessment, functional communication, intensive interaction, music therapy, naturalistic interventions, scripting, sign language, time delay and video modelling, of which 10 were EBPs.

Table 2 demonstrates teachers’ mean knowledge and use of practices, and the percentage of teachers reporting using the practice daily (EBP = evidence-based practice; US = unsupported practice). For knowledge, above 3 indicates “some” knowledge of the practice, and for use, above 3 indicates using the practice one or two times per week.

**Table 2:** Overview of teacher’s knowledge and use of practices

Practice	EBP Status*	Knowledge of EBPs *M (SD)*	Use of EBPs *M (SD)*		% Use Daily
Antecedent Interventions	EBP	2.52 (1.32)		2.37 (1.37)	19.7
ABA	NA	2.23 (1.20)		1.82 (1.09)	8.7
Cognitive Behavioural Interv	EBP	2.85 (1.13)		2.59 (1.26)	23
Developmental Interventions	US	1.75 (1.01)		2.59 (1.26)	23
DRI/A/O	EBP	1.88 (1.19)		1.78 (1.13)	8.7
Discrete Trial Teaching	EBP	1.51 (.99)		1.41 (.90)	5.3
Exercise	EBP	**3.29 (1.14)**		**3.34 (1.24)**	43.7
Extinction	EBP	1.62 (1.07)		1.49 (.90)	4.3
Facilitated Communication	US	2.05 (1.17)		1.93 (1.15)	11
Functional Behaviour Assess	EBP	2.09 (1.25)		1.90 (1.15)	11
Functional Communication	EBP	1.85 (1.12)		1.75 (1.02)	8.1
Intensive Interaction	US	1.93 (1.22)		1.92 (1.20)	12.5
Modelling	EBP	**3.16 (1.38)**		**3.16 (1.33)**	41.4
Music Therapy	EBP	2.07 (1.09)		1.88 (1.10)	11.1
Naturalistic Interventions	EBP	1.71 (1.07)		1.67 (1.05)	8.7
Peer Mediated Interventions	EBP	2.17 (1.24)		2.07 (1.17)	13
PECS	EBP	2.93 (1.30)		2.39 (1,33)	20.7
Play Therapy	US	2.52 (1.14)		2.03 (1.14)	10.6
Prompting	EBP	**3.02 (1.30)**		2.99 (1.37)	37.5
Reinforcement	EBP	**3.36 (1.19)**		**3.41 (1.28)**	51
Response Interruption	EBP	2.88 (1.34)		2.77 (1.35)	32.3
Scripting	EBP	2.20 (1.22)		1.95 (1.08)	9.1
Self-Management	EBP	2.52 (1.30)		2.41 (1.26)	19.7
Sensory Integration	EBP	2.87 (1.30)		2.81 (1.36)	33.2
Sensory Rooms	US	**3.33 (1.27)**		**3.18 (1.42)**	42.8
Sign Language	US	2.10 (1.27)		1.68 (1.16)	9.6
Social Stories/ Social Narratives	EBP	**3.59 (1.09)**		**3.44 (1.21)**	50.5
Social Skills	EBP	**3.18 (1.28)**		2.93 (1.37)	36
Structured Play Groups	EBP	2.99 (1.30)		2.84 (1.37)	33.2
Speech and Language Therapy	US	2.63 (1.36)		2.47 (1.38)	26.9
Task Analysis	EBP	2.17 (1.29)		2.00 (1.24)	14.9
Technology	EBP	2.49 (1.28)		2.25 (1.25)	19.7
TEACCH	NA	2.32 (1.43)		2.07 (1.37)	17.8
Time Delay	EBP	1.97 (1.17)		1.94 (1.23)	16.8
Traditional Teaching	US	**4 (1.20)**		**3.35 (1.34)**	48
Video Modelling	EBP	2.25 (1.31)		1.97 (1.22)	15.8
Visual Supports	EBP	**3.96 (1.10)**		2.14 (1.28)	24.1

ABA = Applied Behaviour Analysis, DRI/A/O = Differential Reinforcement of Incompatible/Alternative/Other Behaviour, PECS = Picture Exchange Communication System, TEACCH = Treatment and Education of Autistic and related Communications Handicapped Children

Bolded scores indicate scores above 3, “somewhat knowledgeable” and use “some of the time”

### Differences in Knowledge and Use of EBPs Based on Teacher Characteristics

Teacher characteristics and the differences in knowledge and use of EBPs across teacher characteristics are displayed in Table 3. The data suggests that participants’ knowledge and use of EBPs differed across a number of factors including teacher position, years’ experience with students with autism, CPD training, hours of ITE and CPD received, and access to support professionals. For teacher position, those who were autism class teachers had the highest knowledge and used EBPs most frequently, while GE teachers had the lowest knowledge and used EBPs least frequently. Teachers who had 12–15 years of experience with students with autism had the highest knowledge and use of EBPs, and teachers who had the most (21 + years) and least (less than 3 years) experience had similar low levels of knowledge and use. Teachers who received CPD before teaching children with autism and those who had received additional CPD had higher levels of EBP knowledge and use in comparison with those who had not received training, and those who had received the most amount of hours of ITE training and CPD training were most knowledgeable and used EBPs more frequently. Lastly, those who had access to support professionals more than 7 times per year had the highest levels of knowledge and used EBPs most frequently, and those who had no access to support professionals had the lowest knowledge of EBPs and used EBPs least frequently.

**Table 3:** Teacher Characteristics and Group Differences in Knowledge and Use of EBPs

Characteristic	*n,* %	Knowledge of EBPs		Use of EBPs	
		Mean (SD)	Group Differences	Mean (SD)	Group Differences
Teacher Position			*F* (4, 203) = 6.20, *p* < .001		*F* (4, 203) = 6.71, *p* < .001
General Education Teacher	207 (57.8%)	2.31 (.75)		2.14 (.64)	
Special Education Teacher	62 (17.3%)	2.78 (.87)		2.51 (.73)	
Learning Support Teacher	18 (5%)	2.69 (.58)		2.41 (.57)	
Principal-Teacher	28 (57.8%)	2.82 (.84)		2.54 (.70)	
Autism Class Teacher	43 (12%)	3.02 (.98)		2.80 (.70)	
Education Level			*F* (3, 202) = 2.05, p = .11		*F* (3, 202) = 1.91, p = .13
Bachelors	140 (39.1%)	2.51 (.80)		2.37 (.71)	
Professional Masters in Education	35 (9.8%)	2.23 (.86)		2.01 (.65)	
Postgraduate Certificate/Diploma	117 (32.7%)	2.55 (.88)		2.33 (.71)	
Masters	66 (18.4%)	2.80 (.87)		2.50 (.71)	
Years Autism Teaching Experience			*F* (6, 200) = 3.46, *p* = .003		*F* (6, 200) = 2.59, *p* = .019
Less than 1 year	36 (10.3%)	2.31 (1.03)		2.11 (.84)	
1–3 years	123 (35%)	2.31 (.72)		2.18 (.64)	
4–7 years	84 (23.9%)	2.75 (.72)		2.52 (.62)	
8–11 years	46 (13.1%)	2.68 (.93)		2.41 (.72)	
12–15 years	35 (10%)	3.09 (1.03)		2.69 (.78)	
16–20 years	14 (4%)	2.48 (.66)		2.31 (.66)	
21 + years	13 (3.7%)	2.33 (.80)		2.19 (.86)	
Autism Specific Training					
Received ITE Training			t(185) = -1.47, *p* = .14		t(185) = -.36, *p* = .72
Yes	192 (60%)	2.63 (.84)		2.35 (.72)	
No	128 (40%)	2.44 (.87)		2.31 (.70)	
CPD Before Teaching a Child with Autism			*t*(206) = 2.58, *p* = .01		*t*(206) = 2.58, *p* = .01
Yes	77 (21.8%)	2.84 (.98)		2.58 (.78)	
No	276 (78.2%)	2.48 (.79)		2.28 (.67)	
Additional Autism Specific CPD			*t*(205) = 3.23, *p* = .001		*t*(205) = 2.67, *p* = .008
Yes	235 (67%)	2.69 (.83)		2.44 (.67)	
No	116 (33%)	2.28 (.83)		2.16 (.75)	
Hours of ITE training			*F* (2, 108) = 8.14, *p* = .001		*F* (2,108) = 12.27, *p* < .001
1–3	126 (65.6%)	2.45 (.84)		2.16 (.70)	
4–8	45 (23.4%)	2.79 (.70)		2.56 (.57)	
9 + hours	21 (10.9%)	3.32 (.59)		3.03 (.48)	
Hours CPD Received			*F* (4, 138) = 4.89, *p* = .001		*F* (4,138) = 6.50, *p* < .001
0–5	58 (25.7%)	2.27(.73)		2.08 (.55)	
6–10	38 (16.8%)	2.49 (.88)		2.31 (.71)	
11–20	50 (22.1%)	2.96 (.70)		2.71 (.58)	
21–30	31 (13.7%)	2.70 (.80)		2.30 (.64)	
30 +	49 (21.7%)	2.97 (.84)		2.71 (.66)	
Support from Professionals					
HSE Psychologist*	101 (27.4%)				
Occupational Therapist	192 (52%)				
Speech and Language Therapist	196 (53.1%)				
Behaviour Support Specialist	67 (18.2%)				
NEPS Psychologist*	186 (50.4%)				
CAMHS*	60 (16.3%)				
SESS*	76 (20.6%)				
NBSS*	11 (3%)				
Private Consultants	37 (10%)				
Other	9 (2.4%)				
Access to Supports per Year			*F* (4, 202) = 7.34, *p* < .001		*F* (4, 202) = 8.40, *p* < .001
0 times	84 (25.4%)	2.24 (.80)		2.03 (.65)	
1–2 times	193 (58.3%)	2.55 (.84)		2.36 (.68)	
3–4 times	39 (11.8%)	2.87 (.65)		2,64 (.63)	
5–6 times	11 (3.3%)	3.43 (.61)		2.99 (.47)	
7 + times	4 (1.2%)	3.79 (.51)		3.39 (.57)	

HSE = Health Service Executive, NEPS = National Educational Psychological Service, CAMHS = Child and Adolescent Mental Health Services, SESS = Special Education Support Service, NBSS = National Behaviour Support Service

### Post-hoc Tests

Bonferroni post-hoc tests were conducted to further examine significant differences in knowledge and use of EBPs in the following variables: teacher position, years of experience, hours of ITE and CPD training, and number of times teachers had received support from professionals per year.

For teacher position, there were significant differences between GE teachers (*M* = 2.31, *SD* = 0.75) and special education teachers knowledge of EBPs (*M* = 2.78, *SD* = 0.87; *p* = 0.014), and autism class teachers knowledge of EBPs (*M* = 3.02, *SD* = 0.98; *p* = 0.001). There were no other significant differences between teacher position in knowledge. For use of EBPs, there were significant differences between GE teachers (*M* = 2.14, *SD* = 0.64) and special education teachers use of EBPs (*M* = 2.51, *SD* = 0.73; *p* = 0.03), and autism class teachers use of EBPs (*M* = 2.80, *SD* = 0.70; *p* < 0.001).

For years of experience, there were significant differences in knowledge between those with 1–3 years of experience (*M* = 2.31, *SD* = ,72)and those with 12–15 years of experience (*M* = 3.09, *SD* = 1.03; *p* = 0.005). There were no other significant differences between groups based on experience. There was no significant difference in use based on experience.

There was a significant difference in knowledge between those who had 1–3 h of training in autism in their ITE (*M* = 2.45, *SD* = 0.84)and those who had 9 + hours (*M* = 3.32, *SD* = 0.59; *p* < 0.001). There was also significant differences in use based on autism training in ITE, between those with 1–3 h (*M* = 2.16, *SD* = 0.70) and those with 9 + hours (*M* = 3.03, *SD* = 0.48; *p* < 0.001).

For CPD hours, those who had received 0–5 h (*M* = 2.27, *SD* = 0.73) differed significantly to those who had received 11–20 h (*M* = 2.96, *SD* = 0.70; *p* = 0.006), and those who had received 30 + hours (*M* = 2.97, *SD* = 0.84; *p* = 0.002). The same held true for use of EBPs, with significant differences between those who had received 0–5 h (*M* = 2.08, *SD* = 0.55) compared to 11–20 h (*M* = 2.71, *SD* = 0.58; *p* = 0.001), and those who had received 30 + hours (*M* = 2.71, *SD* = 0.66; *p* < 0.001).

In relation to receiving support from professionals, there were significant differences in knowledge between those who had received no support (*M* = 2.24, *SD* = 0.80) and those who had received support 3–4 times per year (*M* = 2.87, *SD* = 0.65; *p* = 0.021), those who had received support 5–6 times per year (*M* = 3,43, *SD* = 0.61; *p* = 0.003), and those who had received support 7 + times per year (*M* = 3.79, *SD* = 0.51; *p* = 0.003). There was also significant differences between those who had received support 1–2 times per year (*M* = 2.55, *SD* = 0.84) and those who had received support 7 + times per year (*M* = 3.79, *SD* = 0.51; *p* = 0.026).

For use of EBPs, there was significant differences in use between teachers who had received no support (*M* = 2.03, *SD* = 0.65) and those who received support 1–2 times per year (*M* = 2.36, *SD* = 0.68; *p* = 0.023), 3–4 times per year (*M* = ,2.64 *SD* = 0.63; *p* = 0.003), 5–6 times per year (*M* = 2.99, *SD* = 0.47; *p* = 0.004), and 7 + times per year (*M* = 3.39, *SD* = 0.57; *p* = 0.001). Significant differences in use were also found between teachers who received support 1–2 times per year (*M* = 2.36, *SD* = 0.68) and 7 + times per year (*M* = 3.39, *SD* = 0.57; *p* = 0.025).

## Discussion

This paper addresses an important gap in our understanding of how knowledge and use of autism EBPs can be impacted in school settings. The findings suggest that more experienced teachers, teachers in small group classrooms (autism class teachers), teachers who have more hours of training and rate their training higher, and who receive more support from multi-disciplinary professionals have more knowledge of EBPs and employ them more frequently. This has implications for our understanding of autism EBP implementation in education settings.

In relation to autism practices, teachers in this sample had low levels of knowledge and use of EBPs. Teachers were most knowledgeable of traditional teaching methods which was categorised as an unsupported practice, and knowledgeable to some extent of seven EBPs, including; visual supports, social stories/ social narratives, exercise, modelling, prompting, reinforcement and social skills training. This is reflective of prior research (Barry et al., 2020b; Finlay et al., 2019) in which teachers reported using visual supports widely with students with autism. Teachers reported very little knowledge of six EBPs, including functional communication training, discrete trial teaching, peer-mediated instruction, time delay, technology aided instruction, self-management, parent-implemented interventions, and video modelling, representing over one-fifth of all EBPs identified by Steinbrenner et al., (2020). Teachers in this study also reported low levels of use of EBPs. Four EBPs (exercise, modelling, reinforcement and social stories/ social narratives) were used at least some of the time, with social stories/ social narratives being used most frequently.

In comparison with other contexts, teachers in this sample had very low levels of knowledge and use of EBPs, giving some preliminary evidence for the differences in EBP use across countries and contexts. The teachers in this sample reported using four EBPs “some of the time”, which was less than the 7 EBPs reported in an Australian study using the same measure (Sulek et al., 2019). Furthermore, half of the teachers in this sample reported using only 2 EBPs daily, in comparison with a study from the USA where half of the teachers reported using 7 EBPs daily (McNeill, 2019). There were also differences in use found between teacher and classroom types. In our study, autism class teachers used EBPs most frequently and GE teachers used EBPs least frequently, similar to McNeill’s (2019) study which found that special educators in self-contained classrooms used EBPs most frequently and general educators used them least frequently. Our findings add weight to the concerns surrounding the contextual fit of autism EBPs for general educators in particular, perhaps because these teachers have more competing demands such as larger groups of students to cater for and other issues like time constraints or non-specialist training (Barry et al., 2020a, 2020b; McNeill, 2019). As recent research indicates that students with autism are more frequently educated in GE classrooms (Hehir et al., 2016; NCSE, 2016), our results highlight the need for further exploration of the implementation processes and adaptations that may be necessary to bridge the research-to-practice gap in a typical classroom (Cook & Odom, 2013; Steinbrenner et al., 2020).

In relation to factors impacting EBP use, the data showed that knowledge of EBPs and use of EBPs were significantly associated, consistent with previous research in school settings and the broader EBP literature (Paynter et al., 2017; Sulek et al., 2019). This adds to the growing support for knowledge as a key factor impacting EBP use which may be contextually independent. As systems of teacher preparation, and teacher continuous professional development varies from country to country our findings indicate that advances in one jurisdiction may not necessarily translate to another jurisdiction. For example teachers’ knowledge and use of EBPs also differed based on their training and rating of training, and so the low levels of knowledge and use of EBPs in this sample is perhaps reflective of the system of teacher preparation and continuous professional development in Ireland. Over 65% of teachers in this study report receiving less than 3 hours of autism-specific training in their ITE, with 36% of teachers reporting receiving no ITE or CPD training at all. Furthermore, 78% of teachers reported receiving no autism-specific CPD training before beginning to teach a child with autism, and 33% of teachers report receiving no additional CPD training at all. While these findings highlight how lack of preparation to implement EBPs impact on autism EBP use, it is also reflective of the broader literature which finds that in many countries teachers in general receive minimal preparation for educating students with special educational needs (Hick et al., 2018), and professional development programmes are often not impactful in developing teacher’s skills (Fennell & Dillenburger, 2018). This indicates that an integrated international approach to preparing teachers to support students with autism is warranted in contrast with the fragmented approach currently in place.

Much training in EBPs consists of stand-alone workshops on specific interventions or topics (Alexander et al., 2015), which has been shown less efficacious than training consisting of coaching and mentoring. Furthermore, in order for teachers to be able to effectively understand and implement EBPs, a certain level of research literacy is required, whereby teachers can critically examine the academic literature and put this research into practice (McGann et al., 2020). Emphasising the importance of coaching and mentoring (Alexander et al., 2015), this study also finds evidence for the significance of professional supports in aiding teachers develop capacity for implementing EBPs with students with autism, as those who had received more support reported more frequent use of EBPs. However, the results of this study also suggest that teachers receive very little support from other professionals, with 80% of teachers receiving support from professionals two or less times per year, and a quarter of all teachers reporting no access to professionals at all.

Thus, overcoming the research-to-practice gap may require a number of approaches as suggested by the data in this study. First, teacher preparation courses may be better placed to educate teachers to critique research and to identify appropriate interventions which they can implement and intensify if necessary, therefore developing teachers capacity to act as research-based practitioners (McGann et al., 2020). Secondly, given the importance of mentoring and coaching and the existence of systems of collaboration already extant in schools (Barry et al., 2020b; Daly et al., 2016), developing systems of mentoring and communities of practice (Hall, 2015) may be a socially valid solution to provide increased supports to teachers. Previous research has demonstrated the positive effects of train-the-trainer models (Shire & Kasari, 2014), whereby there is a designated expert in schools who then facilitates the training and upskilling of other teachers. Exploring the utility of this type of peer-support and collaboration may be an avenue worth exploring in contexts where teachers struggle to access training and support. Finally, a collaborative international approach to preparing teachers in best practice to support students with autism is warranted. A recent study by Morin et al (2020) examined the efficacy of an online training course in increasing school professionals knowledge of EBPs (Autism Focused Intervention Materials and Resources; AFIRM), and found that the modules were effective at increasing autism EBP knowledge in school professionals from a large number of countries. However, a shortcoming of this study was that no measure of whether this increase in knowledge translated into an increase in EBP use in classrooms was taken, and as such it remains unclear if contextual adaptations need to be made to these types of resources, or if increased implementation support may be needed in some contexts. Research on teacher training that can be adapted to context and take account of local idiosyncrasies whilst providing high quality information, training and support would ensure that students with autism benefit from state of art intervention wherever they may be geographically located.

### Limitations

This study was a cross-sectional study, and due to bias of responding whereby those with a stronger interest in responding are possibly more likely to partake in research, there is potential for this sample to be negatively skewed. However, the sample had geographical spread, with participants from all 26 counties in the Republic of Ireland, and the findings are broadly in line with prior Irish research which has indicated poor training and knowledge in teachers (Anglim et al., 2018; Finlay et al., 2019).

## Conclusion

This paper adds significantly to our understanding of how teachers knowledge and use of autism EBPs can differ based on teacher characteristics. Overall, it would appear that teachers in our study received little training in their ITE, and have differential access to CPD. Access to additional CPD in particular was identified as significant in the use of EBPs, and as many GE teachers will now have a child with autism included in their classroom, access to timely appropriate CPD is imperative for these teachers. Support from allied professionals is also central to teachers feeling supported and confident in using autism EBP’s. Based on our findings teachers would also benefit from receiving mentoring or training from special education teachers, and as strong systems of collaborative practice appear to be a feature in autism education (Barry et al., 2020b; Daly et al., 2016), these could be capitalised upon by schools and professionals to provide an effective, evidence-based approach to teaching students with autism.
